# A new species of *Astreptolabis* in mid-Cretaceous amber from northern Myanmar, with the discovery of the first male of Astreptolabidinae (Dermaptera)

**DOI:** 10.3897/zookeys.911.38845

**Published:** 2020-02-12

**Authors:** Yue Mao, Michael S. Engel, Dong Ren, Taiping Gao

**Affiliations:** 1 College of Life Sciences, Capital Normal University, 105 Xisanhuanbeilu, Haidian District, Beijing 100048, China; 2 Academy for Multidisciplinary Studies, Capital Normal University, 105 Xisanhuanbeilu, Haidian District, Beijing 100048, China; 3 Division of Entomology, Natural History Museum, and Department of Ecology & Evolutionary Biology, 1501 Crestline Drive – Suite 140, University of Kansas, Lawrence, Kansas 66045-4415, USA; 4 Division of Invertebrate Zoology, American Museum of Natural History, Central Park West at 79th Street, New York, New York 10024-5192, USA

**Keywords:** Cenomanian, earwigs, male genitalia, Neodermaptera, Pygidicranidae, taxonomy

## Abstract

A new species of one of the basal families among extant Dermaptera, Pygidicranidae, is described from mid-Cretaceous amber of Myanmar based on two females and a male. *Astreptolabis laevis***sp. nov.**, belongs to the extinct subfamily Astreptolabidinae, sharing the diagnostic combination of features typical of this group, such as the well-developed compound eyes, large pronotum, and straight and tubular cerci. The discovery of a male with its genitalia partly exerted permits characterization of traits for the subfamily and provides further information on the uniqueness and affinities of the subfamily. In addition, the extended hind wing allows for a comparison between the folding mechanism between these fossils and their modern counterparts, demonstrating considerable conservatism in hind wing evolution among Dermaptera.

## Introduction

Earwigs (order Dermaptera) are one of the smaller orders of insects and consist of approximately 2000 modern species segregated into 12 families (Engel and Haas 2007; Engel et al. 2016). These are characteristic insects, with their generally flattened appearances, often leathery integument, tegminized forewings, broad fan-shaped hind wings, and, most distinctive of all, the terminal forceps formed of their modified cerci (Grimaldi and Engel 2005).

Within the Dermaptera, the modern fauna falls entirely within the suborder Neodermaptera (Engel 2003; Grimaldi and Engel 2005; Engel and Haas 2007), with the most basal families falling into the infraorder Protodermaptera. Protodermaptera include the families Diplatyidae, Haplodiplatyidae, Karschiellidae, and Pygidicranidae, all of which plesiomorphically possess equal-sized ventral cervical sclerites, often carinate femora, and in the most basal members a segmented pygidium (Popham 1985; Engel 2003), among other features of the male genitalia. Presently, the earliest definitive Neodermaptera are found in the Lower Cretaceous (Engel et al. 2002, 2011; Engel and Chatzimanolis 2005; Engel and Haas 2007), and there have been 22 taxa described from Cretaceous amber. Of these 22 taxa, six are classified in the family Pygidicranidae, including four adults and two nymphs. Although the record of earwigs preserved in Cretaceous and Cenozoic amber has grown rapidly (e.g., Engel 2009, 2011, 2016, 2017; Perrichot et al. 2011; Ross and Engel 2013; Engel and Perrichot 2014; Engel and Grimaldi 2014; Engel et al. 2011, 2015, 2017; Ren et al. 2017, 2018), well-preserved specimens of adult earwigs are still rather uncommon and it remains a difficulty to associate nymphs with adults when not found as syninclusions. Moreover, the precise phylogenetic placement of many fossil earwigs continues to be poorly understood.

Herein, based on three new specimens from the Upper Cretaceous amber of northern Myanmar, a new species of the extinct pygidicranid subfamily Astreptolabidinae is described and figured. As one of the specimens is a male with its genitalia partly exerted, this discovery also permits an account of the male for the subfamily, providing new characters which emphasize the distinctiveness of this lineage. Based on the new species, the diagnosis of the subfamily is slightly emended to accommodate variations previously unknown.

## Materials and methods

The three amber specimens discussed in this study were collected from mines in the Hukawng Valley of Kachin in northern Myanmar. The amber mines are located at the north end of Noije Bum that is at approximately 26.150N, 96.340E, 18 km southwest of Tanai (Shi et al. 2012). The age of Burmese amber is documented as 98.79 ± 0.62 Ma (Shi et al. 2012), which places it precisely at the mid-Cretaceous, in the lowermost Cenomanian near the Albian boundary (Shi et al. 2012; Grimaldi and Ross 2017). The type specimens are housed in the fossil insect collection of the Key Lab of Insect Evolution and Environmental Changes, College of Life Sciences, Capital Normal University, Beijing, China.

The new specimens were examined and photographed using a Leica M205C dissecting microscope with a Leica DFC450 digital camera system. The detailed and enlarged photos were taken by using a Nikon SMZ 25 microscope with a Nikon DS-Ri 2 digital camera system. Line drawings were prepared by using Adobe Illustrator CC and Adobe Photoshop CS5 graphics software. Morphological terminology and the higher classification follow those of Engel and Haas (2007), Giles (1963), and Hincks (1956). The description is based on that of the holotype female and paratype male, with differences in the paratype female discussed separately.

## Taxonomy

### Family Pygidicranidae Verhoeff, 1902

#### Subfamily Astreptolabidinae Engel, 2011

*Emended diagnosis* (modified from Engel (2011)). Tiny earwigs (ca. 3.50–5.30 mm in length); somewhat dorsoventrally compressed, setation variable (either sparsely setose or minutely hirsute); integument somewhat matt. Head prognathous, broad, slightly broader than anterior border of pronotum, apparently mildly tumid, posterolateral corners gently curved, posterior border not concave; compound eyes well developed, prominent; ocelli absent; antenna with at least 14 antennomeres (as noted by Engel (2011)), scape stout, pedicel longer than wide, flagellomeres longer than wide, progressively more elongate from flagellomere II–X, with X–IV subequal in size. Pronotum large, anterior and posterior borders gently convex, lateral borders slightly divergent posteriorly and rounded, anteriorly slightly narrower than head, posteriorly broader than head, all borders not carinate. Tegmina present, without venation, symmetrical, elongate, outer margins convex, apex gently curved and tapering to midline (not truncate), covering first four abdominal segments; hind wings present, with squama slightly exposed from under tegmina. Femora apparently not carinate; tarsi trimerous, second tarsomere shortest or as long as third tarsomere, not extending beneath base of third tarsomere; pretarsal ungues simple; arolium vestigial. Abdomen slender, elongate (eight visible segments for females), most segments only slightly wider than long, apicalmost segment with straight apical margin, without tubercles. Cerci symmetrical, straight, tubular, gently tapering to acute apex, without tubercles, dentition, or serrations; pygidium not evident. Female valvulae scarcely evident apically, largely hidden; male with stout parameres, apically pointed, without accessory teeth or incisions; two virgae present, both directed apically; each distal lobe with a ventral sclerotized accessory structure bearing a comb of prominent teeth below each virga.

##### Genus Astreptolabis

Taxon classificationAnimaliaDermapteraPygidicranidae

Engel, 2011

5622C3B9-F8DB-5D25-954A-7AC4E59B23A1

###### Diagnosis.

Refer to that of subfamily (*vide supra*).

###### Comments.

Given that the subfamily contains a single genus, *Astreptolabis* Engel, 2011, the diagnosis of the subfamily and genus are identical.

The genus presently includes only two species: the type species, *Astreptolabis ethirosomatia* Engel, 2011, and *Astreptolabis laevis* sp. nov.

##### Astreptolabis laevis sp. nov.

Taxon classificationAnimaliaDermapteraPygidicranidae

D429C2E5-F4D3-5F32-88E7-62F081FA4D40

http://zoobank.org/CFBEA9C6-7BF4-49A8-82AD-7D671B82634F

Figs 1 , 2 , 3 , 4

###### Diagnosis.

The new species can be distinguished from *A. ethirosomatia* on the basis of the more sparse setation, particularly on the head, pronotum, and tegmina (distinctly and minutely hirsute in *A. ethirosomatia*); the larger compound eyes, which encompass the entire lateral surface of the head from the antennal articulations to the posterior border (in *A. ethirosomatia* the compound eyes are smaller, distinctly separated anteriorly from the antennal base and posteriorly from the temple margin); distance between compound eyes subequal to compound eye length (distance between compound eyes in *A. ethirosomatia* distinctly greater than compound eye length); absence of ocular setae (present in *A. ethirosomatia*). On the surface there would appear to be further proportional differences between the new species and the type species, but the holotype of *A. ethirosomatia* is poorly preserved and largely compressed with considerable taphonomic distortion (Engel 2011).

###### Description.

**Figure 1.**
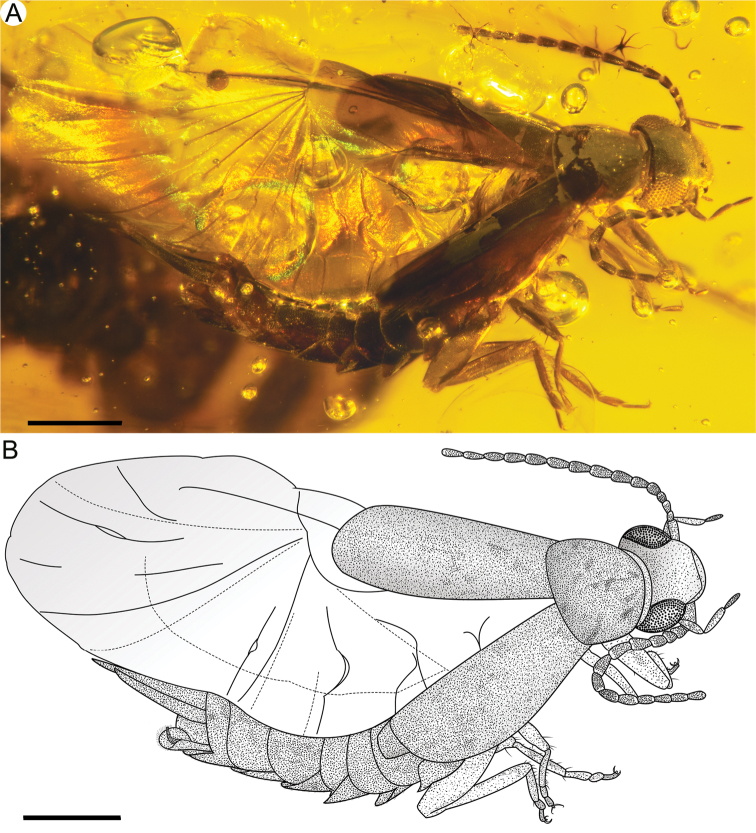
*Astreptolabis laevis* sp. nov., holotype, CNU-DER-MA2018001 **A** photo **B** line drawing. Scale bars: 0.5 mm.

**Figure 2.**
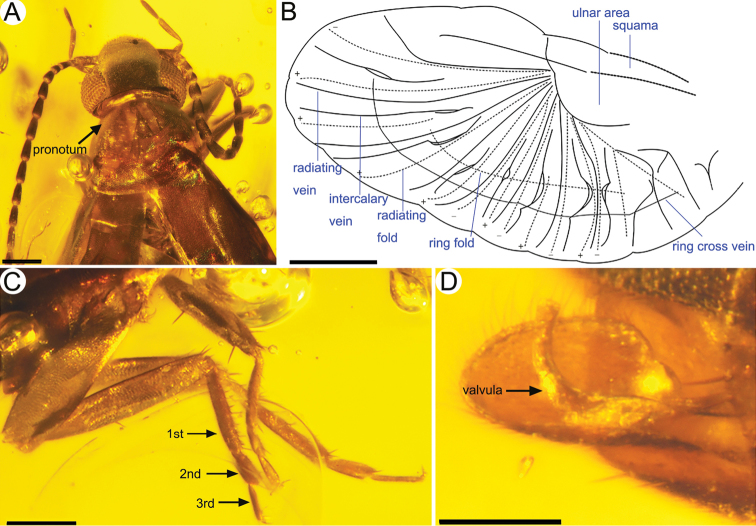
*Astreptolabis laevis* sp. nov., holotype, CNU-DER-MA2018001 **A** photo of pronotum **B** line drawing of hind wing **C** photo of legs **D** photo of vavula. Scale bars: 0.2 mm (**A, C**), 0.5 mm (**B**), 0.1 mm (**D**).

***Female***: Total length as preserved (including cerci) ca. 3.61 mm (Fig. 1); sparsely setose; head medial length from clypeal apex to posterior border 0.47 mm, maximum width (across level of compound eyes) 0.59 mm; compound eye length 0.25 mm, separated from posterior border of head by minute distance. Pronotum medial length 0.51 mm, anterior width 0.37 mm, posterior width 0.60 mm (Fig. 2A); tegmen length 1.18 mm, maximum width 0.46 mm. Abdominal length as preserved (excluding cerci) 1.43 mm, maximum width 0.54 mm; second tarsomere shortest but almost as long as third tarsomere; arolium vestigial; cercal forceps length 0.61 mm, basal width 0.07 mm, separation between bases 0.05 mm. Integument as preserved dark brown, punctate, somewhat smooth throughout. Legs without spines or bristle-like setae (Fig. 2C). Valvulae extending slightly beyond apex of subgenital plate (Fig. 2D).

Hind wings well developed (Fig. 1); area of hindwing 0.5 mm^2^ folded, 2.9 mm^2^ unfolded; squama sclerotized, extending a little beyond apex of tegmina; ulnary area distad squama; eight radiating veins and eight intercalary veins in anal area, with concave and convex folding lines between them; ring fold running through anal fan, intersecting with radiating and intercalary veins in broadened areas (Figs 1, 2B).

***Male***: Total length as preserved (including cerci) ca. 5.30 mm (Fig. 4A, B); sparsely setose; head medial length from clypeal apex to posterior border 0.47 mm, maximum width (across level of compound eyes) 0.71 mm. Pronotum medial length 0.61 mm, anterior width 0.46 mm, posterior width 0.72 mm; tegmen length 1.53 mm, maximum width 0.64 mm. Abdominal length as preserved (excluding cerci) 1.96 mm; second tarsomere shortest but almost as long as the third; arolium vestigial (Fig. 4C); cercal forceps length 0.74 mm. Integument as preserved brown, somewhat smooth throughout. Legs without spines or bristle-like setae. Parameres broad, tapering to acute apex, without incisions or teeth, with a series of sensory setae along inner margin; two virgae extended, apically, with comb-like accessory sclerites positioned ventrally on distal lobes (Fig. 4D, E).

Hind wing well developed, congruent with the description above, and unfolded.

**Figure 3.**
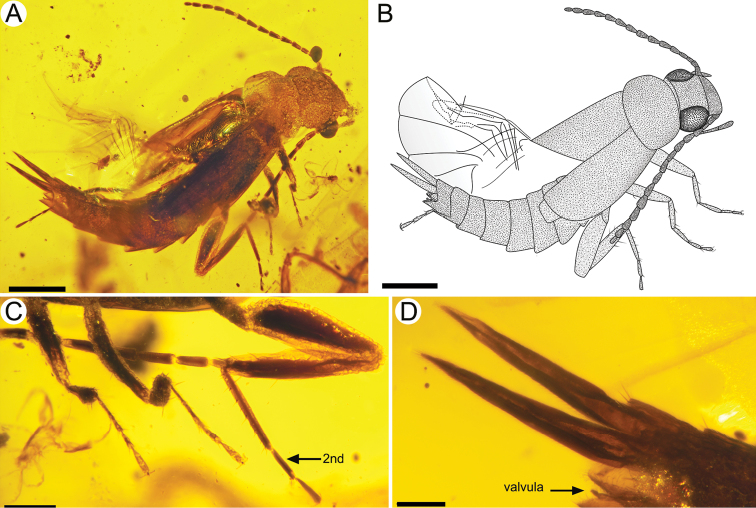
*Astreptolabis laevis* sp. nov., paratype, CNU-DER-MA2018002 **A** photo **B** line drawing **C** photo of legs **D** photo of cerci. Scale bars: 0.5 mm (**A, B**), 0.2 mm (**C**), 0.1 mm (**D**).

**Figure 4.**
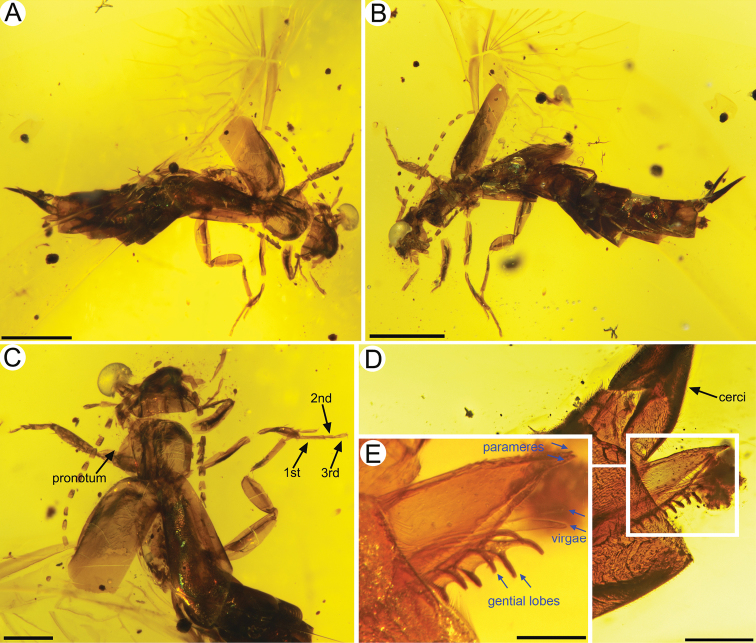
*Astreptolabis laevis* sp. nov., paratype, CNU-DER-MA2018003 **A** dorsal view **B** ventral view **C** photo of pronotum and legs **D** photo of male genitalia **E** enlarged view of male genitalia. Scale bars: 1 mm (**A, B**), 0.5 mm (**C**), 0.2 mm (**D**), 0.1 mm (**E**).

###### Remarks.

The hind wing of the holotype of *A. laevis* is well preserved, and one is unfolded and extended. The base of the hind wing is obscured because of the position of the specimen, but most of the preserved structures are similar to those of extant earwigs. The female paratype CNU-DER-MA2018002 shares the same characters with the holotype, but the integument of this paratype is somewhat roughened and the pronotum seems broader than the holotype; however, these differences may be the result of taphonomy. The male paratype CNU-DER-MA2018003 shares the same characters with the holotype except for a larger body size, which seems to be a sexual difference. Otherwise, differences in body size are mainly reflected in tegmen length and abdominal length which are longer than the female, but otherwise proportional.

###### Type material.

Holotype, ♀, CNU-DER-MA2018001, dorsal view, a well-preserved complete female. Paratype ♀ CNU-DER-MA2018002, dorsal view, a well-preserved complete female. Paratype ♂, CNU-DER-MA2018003. All type material deposited in College of Life Sciences, Capital Normal University, Beijing, China.

###### Locality and horizon.

Hukawng Valley, Kachin State, northern Myanmar; lowermost Cenomanian, mid-Cretaceous.

###### Etymology.

The specific epithet is the Latin word *laevis*, meaning, “polished” or “smooth”, in reference the integumental surface of the species.

## Discussion

Up to now, many than 1000 species of insects have been reported from Burmese amber (Grimaldi et al. 2002; Ross 2019), including termites (Engel et al. 2016; Zhao et al. 2019), stick insects (Chen et al. 2018, 2019), scorpionflies (Lin et al. 2019), lots of wasps (Zhang et al. 2018), beetles (Cai et al. 2018), lacewings (Liu et al. 2018), etc.; however, earwigs are still quite rare compared to most of the other groups. *Astreptolabis* are the smallest earwigs within Pygidicranidae so far, females being as small as about 3.5 mm in length including cerci. Antenna has at least 14 antennomeres, which is an unusually small number for basal Neodermaptera and likely autapomorphic for the subfamily. Although the known representatives are quite peculiar among living and fossil earwigs, particularly in the straight, tapering, tubular cerci which clearly would have had little force as a grasping structure (contrary to virtually all other Neodermaptera), it has the usual traits typical of the suborder such as the absence of ocelli, the trimerous tarsi, unsegmented cerci, and absence of venation in the tegmina (Engel 2011). The straight and tubular cerci which likely did not function for grasping could imply that astreptolabidines used other strategies for predation, or were scavengers or detritivores and therefore did not hunt. The new species is typical in nearly all traits with the type species, aside from minor differences in largely setation, and proportions of structures. Nonetheless, the new species helps to refine our understanding of the circumscription for the genus and subfamily, and gives us some initial knowledge as to variations that may occur within the lineage. Unfortunately, while it would be revealing to learn more about the structure of the ventral cervical sclerites and thoracic sterna, these cannot be discerned in any of the new specimens and await future discoveries to provide such insights. Nonetheless, the discovery of the male for the group is important, and the uniqueness of the Astreptolabidinae is reinforced by the peculiar features of the male genitalia. Like other Pygidicranidae, the male has two virgae, rather than four terminal virgal sections present in Diplatyidae. However, unlike most other Pygidicranidae the parameres are broader and lack terminal teeth or incisions, and are instead comparatively simple, tapering to an acute apex. These are either specializations apomorphic for the subfamily, or could also suggest that including Astreptolabidinae within Pygidicranidae renders the family paraphyletic. If the latter, then there may be need to elevate the subfamily to family rank (as Astreptolabididae Engel, *nomen translatum*), but such a formal decision must await further character data such as the form of the thoracic sterna which could provide evidence of affinity to one or more subfamilies within Pygidicranidae as currently defined. However, the female valvulae slightly extending beyond the subgenital plate is a trait known only among the Pygidicranidae (Engel and Grimaldi 2004), and this feature in *A. laevis* tends to corroborate its inclusion among pygidicranids. For the time being, the male of *A. laevis* emphasizes the distinctiveness of the lineage and highlights the need to obtain further character data for this group of peculiar, ancient Neodermaptera. If the subfamily were to fall outside of Pygidicranidae it remains uncertain whether it would be basal to the family or more closely related to Epidermaptera, and perhaps information on sternal forms would aid such a determination. The presence of complex, heavily sclerotized accessory structures ventral to the paired distal lobes is at least reminiscent of the accessory structures sometimes found among diplatyids, although the size and form of those in *Astreptolabis* are drastically different. If these were homologous, then it might suggest that Astreptolabidinae are intermediate in phylogenetic position between the basal families Diplatyidae, Haplodiplatyidae, and Karschiellidae relative to Pygidicranidae.

To date, there are only four Cretaceous amber species of adult pygidicranids published: *Burmapygia resinata* Engel & Grimaldi, 2004, *A. ethirosomatia* Engel, 2011, *Stonychopygia leptocerca* Engel et al., 2017, and *Gracilipygia canaliculata* Ren et al., 2017 (Engel and Grimaldi 2004; Engel et al. 2017; Ren et al. 2017). As noted by the features of the subfamily (*vide supra*), *Astreptolabis* differs greatly from all of these groups.

Interestingly, the wing morphology of the hind wings of *A. laevis* is quite similar to extant earwigs. Though the broad attachment and the base of the hind wing is covered by the tegmina, the anal area is relatively clear, and the same areas of folding can be discerned as is found across all Neodermaptera, emphasizing the consistency of this specialization within the order. In addition, the shortened tegmina is known to allow for flexibility in the abdomen and its role in folding the hind wings when not in use and this behavioral repertoire is likely also conserved.

## Supplementary Material

XML Treatment for Astreptolabis

XML Treatment for Astreptolabis laevis
